# *ABCC8*-Related Maturity-Onset Diabetes of the Young (MODY12): A Report of a Chinese Family

**DOI:** 10.3389/fendo.2020.00645

**Published:** 2020-09-11

**Authors:** Leweihua Lin, Huibiao Quan, Kaining Chen, Daoxiong Chen, Danhong Lin, Tuanyu Fang

**Affiliations:** Department of Endocrinology, Hainan General Hospital, Hainan Affiliated Hospital of Hainan Medical University, Haikou, China

**Keywords:** *ABCC8* gene, missense mutation, MODY, diabetes, metformin

## Abstract

Maturity-onset diabetes mellitus of the young (MODY) is a monogenic diabetes characterized by autosomal dominant inheritance. Its atypical clinical features make diagnosis difficult and it can be misdiagnosed as type 1 or type 2 diabetes. Fourteen subtypes of MODY have been diagnosed so far, of which MODY12 is caused by mutation of the *ABCC8* (ATP Binding Cassette Subfamily C Member 8) gene, which is rarely reported in China. This paper reports a Chinese family of MODY12 caused by a rare missense mutation on the *ABCC8* gene, which has not been reported to be associated with MODY in China or in other countries, with the aim of increasing clinicians' awareness and attention to the disease.

## Introduction

Monogenic diabetes refers to a specific type of diabetes resulting from monogenic mutation, of which the most common type is maturity-onset diabetes of the young (MODY). MODY is an autosomal dominant hereditary disease leading to dysfunction of the pancreatic β-cells. Since its discovery, mutations have been identified for at least these 14 genes (*HNF4A, GCK, HNF1A, PDX1, HNF1B, NEUROD1, KLF11, CEL, PAX4, INS, BLK, ABCC8* [ATP Binding Cassette Subfamily C Member 8], *KCNJ11, and APPL1*) for MODY (1–3). A very high prevalence of family members carry the mutated gene, and patients in the same family have similar clinical manifestations. About 80% of MODY is previously misdiagnosed as type 1 or type 2 diabetes (4–6) because it is a relatively rare condition and there is low awareness of the clinical phenotype and availability of testing. Its atypical clinical features are the main cause of its misdiagnosis, and the diagnosis relies more on genetic testing, which is expensive for the average wage-earner and is not covered by all medical insurance institutions at present. Correct diagnosis of MODY is essential for optimizing treatment, prognosis, and genetic counseling. At present, related research on MODY is relatively scarce in China. Here, we report a family of MODY12, which is caused by a rare missense mutation.

## Family

**Figure 1:**
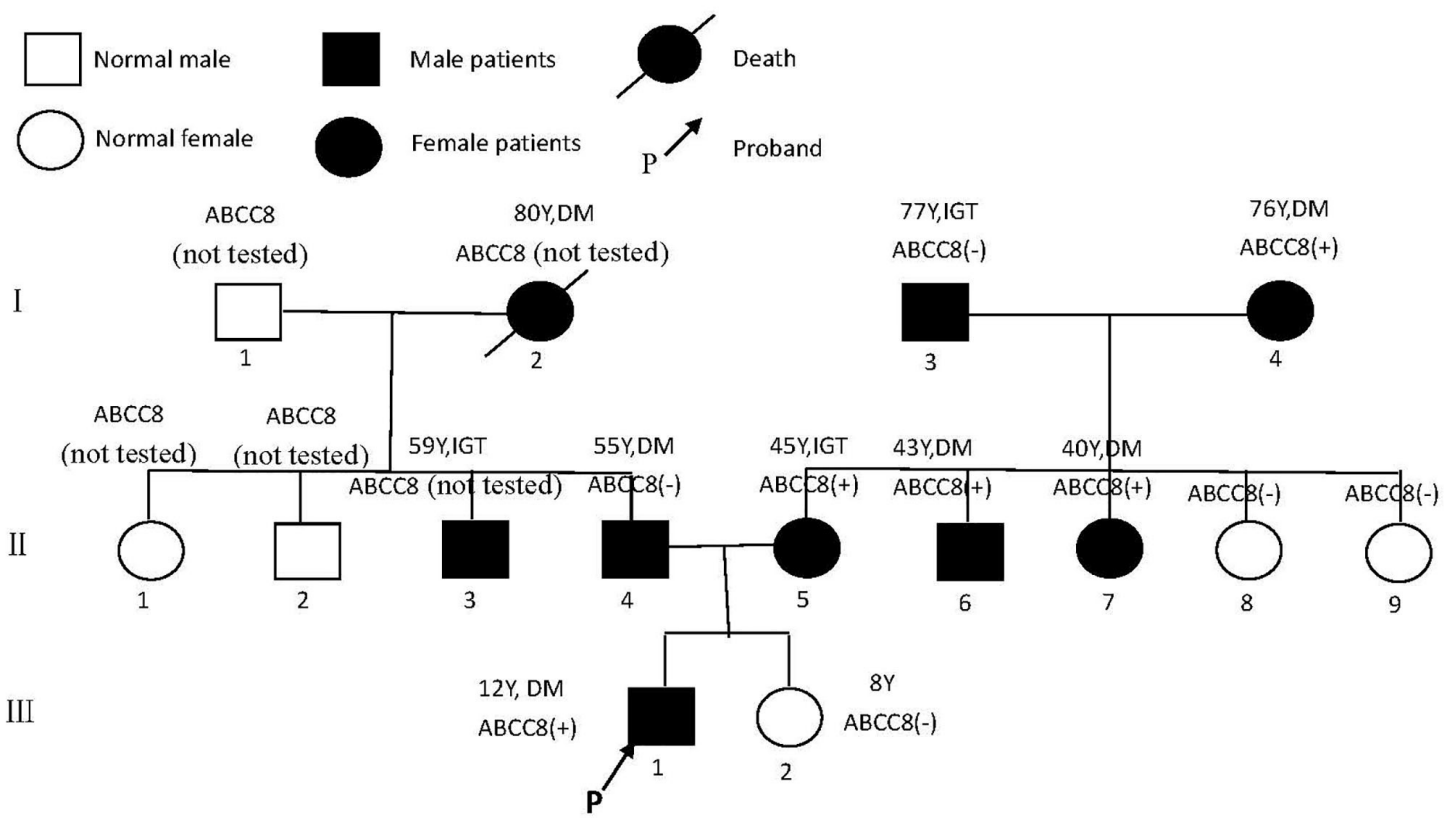
Pedigree of family with diabetes.

The 1999 World Health Organization diagnostic criteria were used to diagnose diabetes mellitus (DM) or impaired glucose tolerance (IGT) (7). The family has a family history of three generations of diabetes (Figure 1). A total of nine people have been diagnosed with diabetes or impaired glucose tolerance, of whom three had been diagnosed before the age of 45 years, and the earliest diagnosed age is 12 years old.

### The Proband (III1)

Male, 12 years old, Han ethnicity. He was admitted to hospital on January 4, 2019, because of polyuria, polydipsia, polyphagia, weight loss for half a year, binocular photophobia, and lacrimation for 2 weeks. There were no obvious causes for the above symptoms, and he had accompanying weight loss of 11 kg for half a year, but had ignored it. Two weeks ago, because of binocular photophobia, tearing, pain, and blurred vision, he visited a local hospital. There, it was found that his fasting blood glucose was 17.1 mmol/L and that he was positive for urine ketone bodies, which was considered type 1 diabetes viral keratitis. He was transferred to our hospital for further treatment.

#### Personal History

He was the first child of a 25-year-old woman with spontaneous term delivery at week 34 of an uneventful pregnancy; his birth weight was 2,700 g. When he was 3 months old, he received hyperbaric oxygen therapy for 4 months because of motor system developmental delay. At 1 year old, his growth and development were satisfactory as children of the same age. His intelligence is normal.

#### Family History

His grandmother was hospitalized when she was 80 years old for pancreatic cancer, where it was found that her blood sugar was elevated, but she eventually died from respiratory failure caused by infection.

#### Physical Examination

Height, 173 cm; weight, 70 kg; body mass index (BMI), 23.39 kg/m^2^, regular pulse (86 beats per min), blood pressure (BP), 125/79 mmHg; waist circumference, 83 cm. He had binocular conjunctival hyperemia and edema. He had no buffalo hump, moon face, skin purple striae, or centripetal obesity. Cardiopulmonary examination showed no abnormality. Biochemical analysis revealed normal liver and kidney function, triglyceride (TG) of 1.16 mmol/L, total cholesterol (CHOL) of 5.82 mmol/L, and low-density lipoprotein cholesterol (LDL-C) of 4.04 mmol/L. His glycated hemoglobin A1c (HbA1c) level was 13.0%. C-peptide (0 min) was 0.438 nmol/L (chemiluminescent; reference range, 0.37–1.47 nmol/L), and C-peptide (120 min) was 1.440 nmol/L. He was negative for anti-glutamic acid decarboxylase (GAD) and anti-insulin autoantibodies. Urine microalbumin was 8.0 mg/L (reference range, 0–30 mg/L). Color ultrasound revealed left popliteal artery intimal–medial thickness and atherosclerotic plaque formation at the proximal part of the right subclavian artery. The optic fundi revealed no hemorrhages or exudates, and the fundal vessels were unremarkable. Neuroelectromyography was normal. He was put on combination therapy with basal (glargine) insulin and recombinant human insulin (RI) to control blood sugar; anti-viral eyedrops and other treatments improved his condition. After 1 week, his glycemic control had improved remarkably, with the vast majority of both fasting (4.2–6.1 mmol/L) and postprandial (6.2–10.0 mmol/L) glucose values within the target range. He had no hypoglycemia reaction, and his review for urine ketone bodies was negative. For better compliance, we changed the insulin combination therapy to mixed recombinant human insulin lispro injection (50R) (12 U daily) after discharge. After 4 months, his body weight gradually increased by about 8 kg (height, 176 cm). On May 16, 2019, we reviewed blood sugar (BS, 0–60–120 min) (no hypoglycemic drugs): 6.10–9.60–11.0 mmol/L, and C-peptide (0–60–120 min): 1.530–3.080–3.130 nmol/L. Biochemical analysis revealed growth hormone of 3.490 ng/ml (reference range, 0.077–10.8 ng/ml). Therefore, we attempted to discontinue insulin therapy and started treatment with 1.0 g/days metformin combined with diet and exercise therapy because of overweight. His glucose fluctuations were within the 6–10 mmol/L range. Reviewing his medical history and adolescent onset, his ketosis had a predisposing factor of viral keratitis, his blood sugar was easily controlled, he had good islet β-cell function, and he was negative for anti-GAD and anti-insulin autoantibodies. Based on the above, MODY was highly suspected. Accordingly, we conducted further investigations on his family.

### The Proband's Father (II4)

He is 55 years old; Han ethnicity. BMI, 28.03 kg/m^2^; waist circumference, 96 cm. BP, 135/82 mmHg. Oral glucose tolerance test (OGTT, 0–120 min): BS, 6.1–11.8 mmol/L; C-peptide, 1.010–4.200 nmol/L. HbA1c, 6.3%. TG, 2.20 mmol/L; CHOL, 5.51 mmol/L; LDL-C, 1.30 mmol/L; liver and kidney function, normal. At present, he is on diet and exercise treatments; his blood sugar is well-controlled.

### The Proband's Mother (II5)

She is 45 years old; Han ethnicity. BMI, 23.18 kg/m^2^; waist circumference, 78 cm. BP, 115/75 mmHg. OGTT (0–120 min): BS, 4.3–10.6 mmol/L; C-peptide, 1.000–4.150 nmol/L. HbA1c, 5.4%. At present, her diabetes is treated with diet and exercise; her blood sugar is well-controlled.

### The Proband's Grandmother (I2)

Deceased; Han ethnicity. When she was 80 years old, she was found to have high blood sugar due to duodenal tubulopapillary adenocarcinoma (late stage). She was not examined further, and was not treated with anti-diabetic therapy. She subsequently died from obstructive jaundice, metabolic encephalopathy, and multiple organ failure.

### The Proband's Uncle (II3)

He is 59 years old; Han ethnicity. BMI, 29.75 kg/m^2^; waist circumference, 98 cm. BP, 135/82 mmHg. OGTT (0–120 min): BS, 5.2–8.6 mmol/L; C-peptide, 1.020–4.760 nmol/L. HbA1c, 6.2%. At present, his diabetes is treated with diet and exercise; his blood sugar is well-controlled.

### The Proband's Uncle (II6)

He is 48 years old; Han ethnicity. He was diagnosed with diabetes 5 years ago, and his diabetes is treated irregularly with glipizide; his blood sugar is generally under control, with no spontaneous ketosis tendency. BMI, 23.18 kg/m^2^; waist circumference, 82 cm. BP, 120/78 mmHg. He refused examination for HbA1c and C-peptide.

### The Proband's Aunt (II7)

She is 40 years old; Han ethnicity. BMI, 22.60 kg/m^2^; waist circumference, 73 cm. BP, 110/70 mmHg. OGTT (0–120 min): BS, 3.6–11.2 mmol/L; C-peptide, 0.571–3.23 nmol/L. HbA1c, 5.5%. At present, her diabetes is treated with diet and exercise; her blood sugar is well-controlled.

### The Proband's Grandmother (I4)

She is 76 years old; Han ethnicity. She has a history of sequelae of cerebral infarction and hypertension for 5 years. BMI, 18.26 kg/m^2^; waist circumference, 69 cm. BP, 110/80 mmHg. OGTT (0–120 min): BS. 4.7–13.2 mmol/L; C-peptide, 0.59–4.51 nmol/L. HbA1c, 5.4%. At present, her diabetes is treated with diet and exercise to control blood sugar and with amlodipine to control BP, both of which are well-controlled.

### The Proband's Grandfather (I3)

He is 77 years old; Han ethnicity. He has had hypertension for 10 years. BMI, 25.96 kg/m^2^; waist circumference, 92 cm. BP, 140/80 mmHg. OGTT (0–120 min): BS, 4.4–9.0 mmol/L; C-peptide, 0.488–3.67 nmol/L. HbA1c, 5.4%. At present, his diabetes is treated with diet and exercise; his blood sugar is well-controlled.

### Genetic Analysis

The combination of negative autoantibodies for type 1 diabetes with inappropriately low C-peptide levels and a family history of diabetes or abnormal glucose tolerance prompted the molecular investigation for MODY. After obtaining informed consent, a venous blood (5 ml) was collected from the proband (III1) as well as his families and placed in an anti-coagulant tube containing ethylenediaminetetraacetic acid (EDTA).

DNA was extracted from the venous blood collected from the patient as well as from the patient's families using the QIAamp DNA Blood Midi kit (Qiagen, Germany), The quantity/quality of the DNA preparations was assessed using a NanoDrop 1000 spectrophotometer (Thermo Fisher, USA).

Exome enrichment was performed using the IDT xGenExome ResearchPanelv1.0 (Integrated DNA Technologies, Coralville, lowa, USA) and 150 base pair, paired end sequencing was performed using an Illumina HiSeq 4000 platform (San Diego, CA, USA). Mean sequencing depth and nucleotides with >20X sequencing depth were 159x and 99.61%, respectively. The raw sequencing reads were aligned by the We-Health BioMedical Technology (Shanghai, China) using the Burrows–Wheeler Aligner (BWA) and SAMtools. Polymorphic variants that showed >0.5% allele frequency in general populations were filtered out (based on the 1000 genomes database). The SIFT, PolyPhen2, MutationTaster were used to assist in predicting the functional impact of identified missense variants (8, 9).

**Figure 2:**
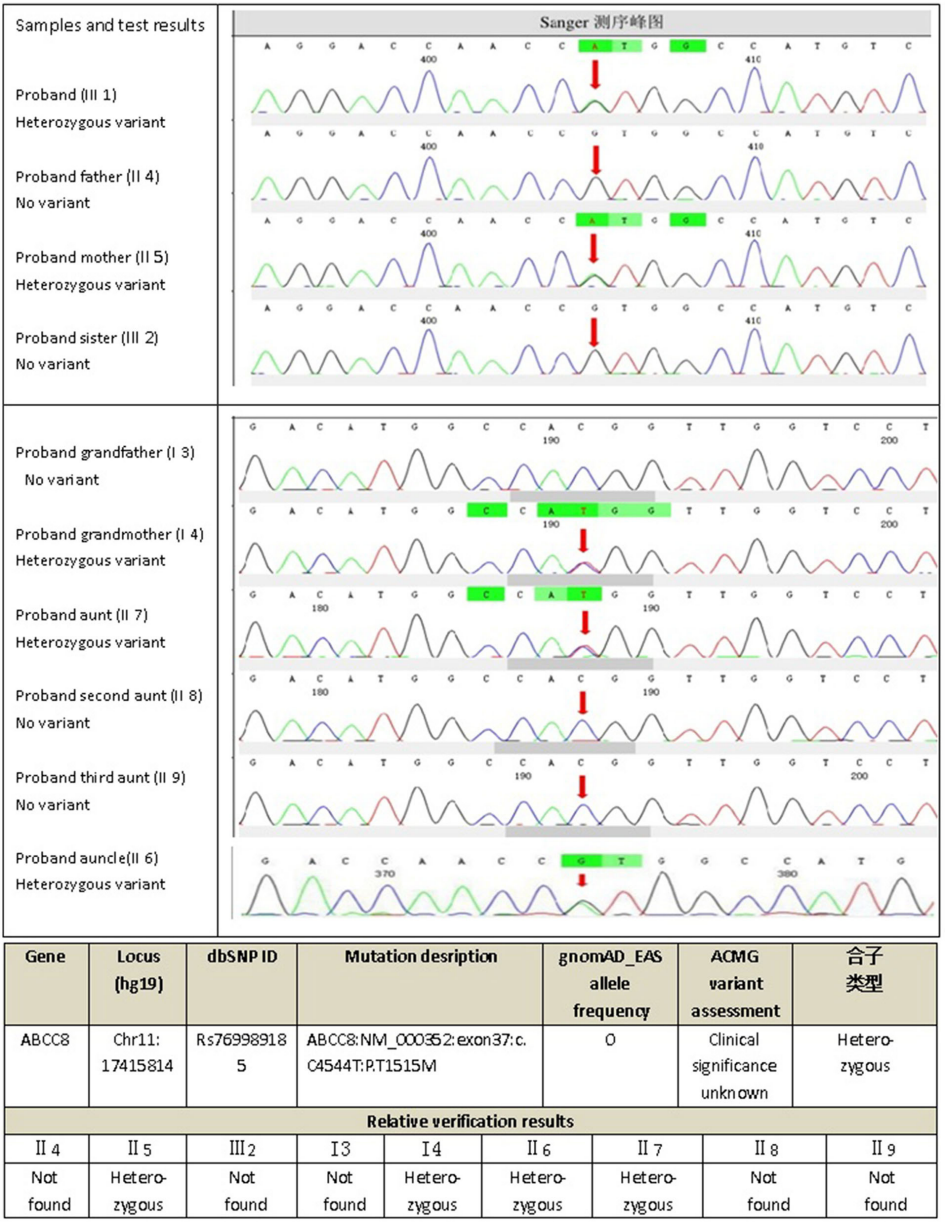
Gene_sequencing results of proband and his family.

The next-generation sequencing data were analyzed for mutations in known MODY-related gene (included the genes for *HNF4A, GCK, HNF1A,PDX1, HNF1B, NEUROD1, KLF11, CEL, PAX4, INS, BLK, ABCC8, KCNJ11, and APPL1*). The sequencing data revealed a heterozygous missense mutation: c.C4544T (p.T1515M), in exon 37 of the *ABCC8* gene of the proband (III1) that was derived from his mother (II5). The amino acid changed from threonine to methionine (Figure 2); no mutations were detected for the remaining 13 genes. For the heterozygous mutation c.C4544T (p.T1515M) of the *ABCC8* gene detected in III1, we verified I3, I4, II4, II5, II6, II7, II8, II9, III1, and III2 using Sanger sequencing and found that III1, II5, II6, II7, and I4 all had the same mutation of the *ABCC8* gene.

Oligonucleotides flanking the genomic locations of identified variantswere designed using the Primer3Plus browser. Polymerase chainreactions (PCR) was performed with the following primers: 5′-CACCCCACAGGACTGAACAG-3′ and 5′- ATCTGCTCCACTCACAGCAC-3′. PCR products were sequenced bi-directionally using Big Dye Terminatorchemistry v3.1 and sequenced using an ABI 3730XL sequencer (AppliedBiosystems/Life Technologies, Carlsbad, CA, USA). Sequences were reviewed manually and compared using CodonCode Aligner to reference sequences: *ABCC8*(NM_000352).

This study was approved by the Ethics Committee of Hainan General Hospital. All subjects gave written informed consent in accordance with the Declaration of Helsinki.

## Discussion

Monogenic diabetes is a special type of diabetes caused by a single gene mutation or defect; it is genetically and clinically heterogeneous, and includes MODY, neonatal diabetes, congenital hyperinsulinemia, and Wolcott-Rallison syndrome (10). First-degree relatives of a MODY family member carrying genetic mutations for the 2 most common MODY subtypes, i.e., GCK-MODY2 or HNF1α-MODY3, could have 95% risk of developing diabetes (11), and patients in the same family are more likely to have similar clinical presentations. MODY is the most common form of monogenic diabetes (1–3, 12, 13). Previous studies agree that the diagnostic criteria of MODY are: (i) autosomal dominant inheritance, (ii) insulin independence within 2 years of onset, (iii) at least one family member diagnosed with diabetes before the age of 25 years, (iv) combined with islet β-cell dysfunction (14).

The identification of a MODY subtype is crucial for the choice of adequate treatment. However, with the maturing of genetic testing technology in recent years, an increasing number of patients with MODY discovered by genetic testing do not fully meet the above diagnostic criteria, and their clinical manifestations tend to be more diverse. Strict adherence to the above criteria may result in missed diagnosis of a large number of patients with MODY. A study from the UK has shown that a significant number of patients with MODY have a primary diagnosis of diabetes and the MODY diagnosis could be confirmed only by performing molecular genetic testing (15), which may lead to inappropriate treatment such as insulin and/or insulin sensitization treatment (16). Therefore, when the onset of diabetes in young patients is not typical type 1 or type 2 diabetes, it should prompt molecular investigation for MODY.

The *ABCC8* gene is located on chromosome 11p15.1 and encodes the sulfonylurea receptor 1 (SUR1) subunit of the ATP-sensitive potassium (K_ATP_) channel in the pancreatic β-cell, which is involved in the electrical activity of the plasma membrane, thereby regulating insulin secretion (17). *ABCC8* gene mutations can cause a variety of phenotypes, resulting in overactivity or underactivity of the K_ATP_ channel, resulting in abnormal glucose metabolism. In 2012, Bowman et al. first reported that MODY12 is caused by *ABCC8* gene mutation, and its clinical manifestations are diverse, may be associated with overweight or obesity, and are usually with no significant hypertriglyceridemia and hypercholesterolemia. Further, such families may also have neonatal diabetic patients (18, 19). As sulfonylureas specifically bind to the SUR1 subunit and shut down the channel to release insulin in a non–ATP-dependent manner, this type of MODY is sensitive to sulfonylureas.

Initially, the present case was misclassified with type 1 diabetes because of his young age and ketosis, despite the negative islet β-cell autoantibodies. The possibility of considering the case as MODY was supported by the presence of diabetes within three generations, the stable C-peptide levels, and the obvious signs of overdose on relatively small insulin doses, which led to overweight. The characteristics of the family are: the proband's age is <25 years old with insulin deficiency, and his ketosis had precipitating factors and was easily eliminated. The diabetes-related antibodies (islet cell antibody [ICA], tyrosine phosphatase-like protein antigen-2 antibody [IA2A], glutamic acid decarboxylase antibody [GADA]) were negative, and a follow-up visit after 4 months on the regimen showed that his glycemic control had remarkably improved, with the vast majority of both fasting and postprandial glucose values within the target range and HbA1c of 5.7%; islet β-cell function was significantly improved, and even the overweight and insulin resistance appeared. All three generations in the family have diabetes or impaired glucose tolerance with autosomal dominant inheritance. In summary, our evidence supports the diagnosis of MODY. Genetic testing verified the presence of the *ABCC8* gene mutation (*ABCC8*: NM_000352: exon37: c.C4544T: p.T1515M) in the proband (III1), his mother (II5) and three maternal relatives (II6, II7, I4) with diabetes or impaired glucose tolerance with similar figures and islet β-cell function, but not in the other relatives (II8, II9, III1) who do not have diabetes or impaired glucose tolerance, suggesting that the above mutation and diabetes have obvious co-segregation in the family. In addition, the proband's uncle (II6) had early-onset diabetes and took glipizide for it without spontaneous ketosis. This mutation (*ABCC8*: NM_000352: exon37: c.C4544T: p.T1515M) can lead to the conserved amino acid residues of this site being replaced by different amino acids throughout the evolution process, resulting in congenital hyperinsulinemia (20), and it has been suggested that congenital hyperinsulinemia caused by *ABCC8* gene mutation can develop into MODY (21, 22), but since then, there has been no report of MODY12 caused by the above mutation. The SIFT score, PolyPhen2 score, and Mutation Taster score was 0, 1, and 1, all suggesting that it is damaging to the protein function. Based on the above predictions, the mutation is considered pathogenic. Therefore, we speculate that this mutation site is the cause of diabetes or impaired glucose tolerance in the proband (III1), his mother (II5), and the three maternal relatives (II6, II7, I4), where the type of diabetes is considered MODY12. I3, II3, and II4 also have diabetes or impaired glucose tolerance, but unlike the proband, they do not have the above mutation, and have characteristics such as central obesity or overweight, insulin resistance, diabetes diagnosed at age >50 years, and whose diagnosis is more prone to type 2 diabetes, which are quite different from the proband (Table 1).

**Table 1:** Clinical characteristics of family members with disease.

	**Gender**	**Age at diagnosis (years)**	**BMI (kg/m^2^)**	**Waistline (cm)**	**0'BS (mmol/L)**	**120'BS (mmol/L)**	**0'CP (nmol/L)**	**HbA1c (%)**
III1	M	12	23.39	83	9.0	18.8	0.438	13.0
II5	F	45	23.18	78	4.3	10.6	0.488	5.4
II6	M	43	23.18	82	-	-	-	-
II7	F	40	22.60	73	3.6	11.2	0.571	5.5
I4	F	76	18.26	69	4.7	13.2	0.59	5.4
II4	M	55	28.03	96	6.1	11.8	1.020	6.3
II3	M	59	29.75	98	5.2	8.6	1.010	6.2
I2	F	80	-	-	-	-	-	-
I3	M	77	25.96	92	4.4	9.0	1.000	5.4

*BMI, body mass index; BS, blood sugar; CP, C-peptide; HbA1c, glycated hemoglobin A1c. The “-” indicates not available*.

The prevalence of MODY subtypes varies widely between countries and ethnic groups. More than 80% of Caucasian patients with MODY are MODY3 or MODY2 (15, 23), but only 7.2–36.7% of Asian patients with MODY are diagnosed as MODY2 or MODY3 (24–27). In China, epidemiological research of MODY is in its infancy, and the distributions of different MODY subtypes are not so clear. Further genes related to MODY are likely to be found, as most patients with MODY have unknown mutations; this group is defined as MODYX. MODYX might be responsible for 80–90% of MODY in China, which is quite different from other populations (25, 26, 28, 29). A lack of awareness of MODY, as well as its similar clinical features to other types of diabetes, means that clinicians cannot often easily distinguish MODY from type 1 or type 2 diabetes without genetic testing, which may lead to misdiagnosis. In the present case, the proband was diagnosed with type 1 diabetes due to early onset (12 years old) with ketosis, the obvious signs of overweight on relatively small doses of insulin, significantly improved islet β-cell function, and even the appearance of insulin resistance. After family investigation and genetic testing, we revised the diagnosis of type 1 diabetes to MODY12, and attempted to discontinue insulin therapy and started treatment with 1.0 g/day metformin combined with diet and exercise therapy. The proband's blood glucose is well-controlled, and he has not gained any more weight. His relatives (II5, II6, II7, I4) with the same *ABCC8* gene mutation as the proband have similar habius, no central obesity, BMI within the 18–24 kg/m^2^ range, mildly elevated blood glucose, and similar islet β-cell function. At present, they only require diet and exercise therapy. Therefore, the identification of a MODY subtype is crucial for the choice of adequate treatment. In the present case, verification of the diagnosis by genetic testing enabled the discontinuation of insulin therapy, which had produced obvious adverse effects, such as increased bodyweight.

In conclusion, we report a case of MODY12 caused by a single nucleotide mutation of cytosine to thymine at position 4,544 of the *ABCC8* gene. This mutation has not been reported to be associated with MODY in China or in other countries. It is a rare missense *ABCC8* gene mutation (dbSNP ID: rs769989185, and has not been reported in ClinVar database: https://www.ncbi.nlm.nih.gov/clinvar/), which might be useful for individualized selection of appropriate treatment and for genetic consulting. The described case also highlights the clinical expression of diabetes related to the *ABCC8* gene and underlines the value of genetic testing in young patients presenting with non-autoimmune diabetes.

## Data Availability Statement

This article contains previously unpublished data. The name of the repository and accession number(s) are not available.

## Ethics Statement

Written informed consent was obtained from the individuals and minor's legal guardian for the publication of any potentially identifiable images or data included in this article.

## Author Contributions

LL collected, analyzed, and interpreted the patient data and was the major contributor to the writing of the manuscript. TF made substantial contributions to the data interpretation, manuscript revision, and conducted the work. HQ helped with manuscript revision in English. HQ, KC, DC, and DL assisted with the patient data analysis. All authors are in agreement with the contents of the manuscript.

## Conflict of Interest

The authors declare that the research was conducted in the absence of any commercial or financial relationships that could be construed as a potential conflict of interest.
